# DDX3X induces mesenchymal transition of endothelial cells by disrupting BMPR2 signaling

**DOI:** 10.1002/2211-5463.70155

**Published:** 2025-11-04

**Authors:** Yu Zhang, Jing Wang, De‐Hui Qian, Yang‐Fan Lv, Tian‐Le Cheng, Da‐Peng Wang, Jing Zhang, Ye Fan

**Affiliations:** ^1^ Department of Respiratory Disease Xinqiao Hospital, Third Military Medical University Chongqing China; ^2^ Department of Cardiology Xinqiao Hospital, Third Military Medical University Chongqing China; ^3^ Department of Pathology Xinqiao Hospital, Third Military Medical University Chongqing China; ^4^ Department of Intensive Medicine Wuxi People's Hospital Affiliated to Nanjing Medical University Wuxi Jiangsu China

**Keywords:** BMPR2, DDX3X, endothelial cell, endothelial‐to‐mesenchymal transition

## Abstract

Endothelial‐to‐mesenchymal transition (EndoMT), a widely recognized biological process leading to abnormal endothelial function, has been implicated in various cardiovascular pathologies. DEAD‐box proteins represent the largest family of RNA helicases associated with multiple physiological and pathophysiological processes; however, their role in the homeostasis of endothelial cells (ECs) remains largely unexplored. Here, we show that the levels of DEAD‐box protein 3 X‐linked (DDX3X), a DEAD‐box RNA helicase protein, were significantly increased during EC transition *in vivo* and *in vitro*. DDX3X overexpression promoted EndoMT as well as endothelial dysfunction and inflammation, whereas its downregulation effectively inhibited this transition in ECs. Mechanistically, elevated DDX3X resulted in downregulation of bone morphogenetic protein receptor type 2 (BMPR2), a protein that is pivotal for maintaining endothelial homeostasis and function. Furthermore, our co‐immunoprecipitation assays demonstrated a molecular interplay between DDX3X and BMPR2. Importantly, DDX3X was shown to promote the lysosomal degradation of BMPR2, thereby interrupting its downstream signal transduction. These findings identify DDX3X as a novel regulator of EndoMT by modulating BMPR2 signaling.

## Graphical abstract

Elevated DDX3X expression led to downregulation of BMPR2, a key regulator of endothelial homeostasis and function. Our co‐immunoprecipitation assays further demonstrated a molecular interaction between DDX3X and BMPR2. Notably, DDX3X promoted lysosomal degradation of BMPR2, thereby impairing its downstream signaling and facilitating endothelial‐to‐mesenchymal transition (EndoMT).

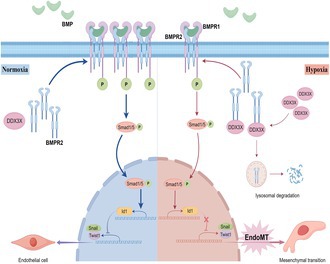

**Abbreviations**
BMPR2: bone morphogenetic protein receptor type 2
BSA: bovine serum albumin
DAPI: 4',6‐diamidino‐2‐phenylindole
DDX3X: DEAD‐box protein 3 X‐linked
DDX5: DEAD‐box protein 5
EC: endothelial cell
EndoMT: endothelial‐to‐mesenchymal transition
FBS: fetal bovine serum
GFP: green fluorescence protein
HUVEC: human umbilical vein endothelial cell
PCR: real‐time polymerase chain reaction
PH: pulmonary hypertension
siRNA: small interfering RNA
SMA: alpha‐smooth muscle actin
TGF‐β: transforming growth factor beta

Endothelial cells (ECs) form the inner lining of blood vessels and are crucial for maintaining vascular homeostasis [1, 2]. Notably, ECs display considerable plasticity, allowing them to undergo endothelial‐to‐mesenchymal transition (EndoMT), a biological process wherein ECs lose their specific markers and functions, acquiring mesenchymal or fibroblast‐like properties [3, 4, 5]. This phenotypic shift is essential during embryonic development and is implicated in multiple cardiovascular disorders, including pulmonary hypertension (PH), atherosclerosis, cardiac fibrosis, and vascular restenosis [6, 7, 8, 9]. Although EndoMT is acknowledged for its role in vascular regulation, the underlying mechanisms driving this process remain incompletely elucidated.

RNA helicases, acting as molecular motors to unwind RNA duplexes, play central roles in nearly all aspects of RNA metabolism [10]. DEAD box proteins represent the largest and most thoroughly studied family of RNA helicases, and their malfunction is associated with a variety of diseases, such as cancer and viral infections [11, 12, 13, 14, 15]. In the context of vascular biology, our prior research demonstrated that RNA helicase DEAD‐box protein 5 (DDX5) has a protective effect on smooth muscle proliferation and systemic vascular remodeling [16]. In addition, human mutations in members of the DEAD‐box protein family are closely linked to vascular conditions, such as Singleton–Merten syndrome and multiorgan venous and lymphatic defect syndrome [17, 18, 19]. More recently, Hang and colleagues reported that activation of DDX5 mediates pericyte dysfunction and promotes intrauterine growth restriction‐related PH, highlighting the multifaceted role of RNA helicases in both systemic and pulmonary circulation [20].

Despite evidence from human and animal studies suggesting the involvement of RNA helicases in vascular pathologies, little is known concerning their role in endothelial homeostasis. In this study, we identified DEAD‐box protein 3 X‐linked (DDX3X), a key member of the DEAD‐box helicases, as a novel regulator of EndoMT by degrading bone morphogenetic protein receptor type 2 (BMPR2), thereby inhibiting its downstream signaling. DDX3X participates in a wide range of biological processes beyond RNA structural remodeling, including cellular stress responses, immune regulation, and embryonic development. Dysregulation of DDX3X has been linked to aberrant inflammatory responses through its modulation of key signaling pathways such as NF‐κB and type I interferon [21]. In the context of vascular biology, emerging evidence also suggests a role for DDX3X in regulating endothelial barrier integrity and mediating responses to vascular injury [22]. In this study, we uncovered a previously unrecognized role for DDX3X in driving EC mesenchymal transition through its interaction with and degradation of BMPR2, providing new insights into the molecular regulation of endothelial homeostasis.

## Materials and methods

### Human sample and animal experiment

Studies involving human lung tissues were conducted in accordance with the principles outlined in the Declaration of Helsinki and were approved by the Institutional Review Boards of the Third Military Medical University in Chongqing, China (AMUWEC20234713). Written informed consent was obtained from all human tissue donors. All animal experiments were conducted following the Guide for the Care and Use of Laboratory Animals, which was reviewed and approved by the Third Military Medical University Animal Care and Use Committee (AMUWEC20210958). As previously described, Sprague–Dawley rats weighing between 200 and 250 grams underwent exposure to hypoxia for three weeks along with an injection of Sugen5416 (20 mg·kg^−1^) (MCE, NJ, USA) [23]. They were then subjected to normoxia for an additional two weeks. In the case of mouse models, eight‐week‐old mice were exposed to chronic hypoxia (10% O_2_) for a period of four weeks.

### Cell culture

Human pulmonary artery endothelial cells were purchased from Lonza, Inc. (Allendale, NJ). Cells were grown in MCDB131 (Gibico, Grand Island, NY, USA) with 10% FBS, 10 ng·mL^−1^ hEGF, and 1% P/S. Human umbilical vein endothelial cells (HUVECs) were purchased from iCELL Biotechnology Co., Ltd (Shanghai, China), cultured in complete endothelial cell culture medium. All cells were grown in 5% CO_2_ at 37 °C and passaged upon reaching confluence.

### Real‐time polymerase chain reaction (PCR)

Samples from tissues and cells were lysed with TRIzol reagent (TaKaRa, Tokyo, Japan) according to the manufacturer's instructions. Then, 1000 nmol of RNA underwent reverse transcription employing the PrimeScript RT reagent Kit with gDNA Eraser (TaKaRa). Real‐time PCR was performed using the SYBR (TaKaRa) on a CFX96 Real‐Time System (Bio‐Rad, CA, USA). Changes in expression levels were analyzed with the comparative Ct method. The specific sequences of the primers used were listed in Table S1.

### Western blot analysis

Protein samples were lysed in RIPA (Beyotime, Wuhan, China) supplemented with 1% phenylmethanesulfonyl fluoride (Beyotime). After measuring the protein concentration using the BCA protein assay kit (Boster, Wuhan, China), the whole protein samples were diluted with 5× SDS/PAGE loading buffer and boiled for 5 min. Equal amounts of protein were loaded on SDS/PAGE gels and transferred onto a polyvinylidene difluoride membrane (PVDF, Millipore, Etten‐Leur, Netherlands). The membranes were incubated with their primary antibodies overnight at 4 °C after blocking with 5% bovine serum albumin (BSA) (Boster) for 1 h at room temperature (Table S2, Dilutions: antibodies against DDX3X [1 : 500], BMPR2 [1 : 500], GAPDH [1 : 1000], eNOS [1 : 500], E‐cadherin [1 : 500], Snail [1 : 500], Vimentin [1 : 500], β‐actin [1 : 1000], ID1 [1 : 500], Smad1/5 [1 : 500], and P‐Smad1/5 [1 : 500]). The blots were washed five times with Tris‐Buffered Saline with Tween‐20 (Beyotime) and incubated with appropriate secondary horseradish peroxidase‐conjugated antibodies (1 : 5000) (Abcam, Cambridge, MA, USA) for 1 h at room temperature. The signals were visualized via enhanced chemiluminescence (Amersham International, Buckinghamshire, UK).

### Lentivirus production and transfection

Scrambled siRNA, DDX3X siRNA (5′‐ACATTGAGCTTACTCGTTA‐3′), BMPR2 siRNA (5′‐AAGCACCGAAGCGAAACTTAA‐3′), and DDX3X overexpression and GFP control constructs were synthesized by Hanbio Biotechnology (Shanghai, China). Lentivirus was used to prepare plasmid vectors that infected cells. In brief, ECs were plated in 24‐well plates at a density of 3 × 10^4^ cells per well and incubated in MCDB131 for 24 h before infection. Following PBS washing, ECs were infected with lentiviruses mixed in endothelial cell complete medium containing 0.5% polybrene (Hanbio, Guangzhou, China). After 24 h, the medium was replaced with fresh complete growing medium, and transfection efficiency (> 95%) was confirmed by observing the expression of GFP under a fluorescence microscope. Real‐time PCR and western blotting were performed for further validation.

### Immunofluorescence

ECs were seeded on the coverslips and fixed with 4% ice‐cold paraformaldehyde for 15 min at room temperature. Then, cells were rinsed with PBS, permeabilized using 0.5% Triton X‐100 and incubated overnight at 4 °C with anti‐DDX3X or anti‐BMPR2 primary antibody. At last, the Cy3‐conjugated and FITC‐conjugated secondary antibodies from Beyotime were added. In all experiments, 4′,6‐diamidino‐2‐phenylindole (DAPI) was used for the counter‐stained nuclei. The cells were visualized using a Zeiss 800 confocal microscope (Zeiss, Jena, Germany). For immunofluorescence analysis of tissues, the procedure is similar to the cell staining. To be brief, the lung tissues were deparaffinized, permeabilized, and blocked. They were then substantially incubated with primary antibodies targeting DDX3X, α‐SMA, and VWF in PBS overnight at 4 °C and with secondary antibodies for 1 h at room temperature. DAPI was used before mounting the coverslip.

### Co‐immunoprecipitation

For immunoprecipitation, cells were lysed with cell lysis buffer (Cell Signaling Technology, #9803, Danvers, MA, USA). Cleared sample solution was obtained by sonication and centrifugation at 12000 ***g*** at 4 °C. The protein concentrations were determined using a BCA kit (Boster). Immunoprecipitation was performed at 4 °C by gently rocking the lysates overnight with either anti‐DDX3X or anti‐BMPR2 antibodies and further incubated with protein A/G magnetic beads (Cell Signaling Technology). The precipitated proteins were washed five times with lysis buffer, boiled in 2× sample loading buffer, and analyzed by western blot. Rabbit normal IgG or mouse normal IgG (Cell Signaling Technology) was used as a negative control.

### Lysosome degradation assay

DDX3X Virus and GFP Virus (Hanbio, Guangzhou, China) Flag‐tagged were transfected into the ECs, grown in 6 plate cell culture dishes and treated with the lysosome inhibitor chloroquine (10 μmol·L^−1^) (MCE) for an additional 8 h. A lysis buffer containing 20 mm Tris–HCl (pH 7.5), 150 mm NaCl, 10 mm EDTA, 1% Triton X‐100, 1% deoxycholate, and a protease inhibitor cocktail (Roche, Basel, Swiss) was used. The cells were lysed via sonication and centrifuged, and the supernatants were collected. After boiling with 5× protein loading buffer, proteins were analyzed by western blot using antibodies specific for BMPR2.

### Cycloheximide chase assay

Cells were treated with 10 mg·mL^−1^ cycloheximide to inhibit *de novo* protein synthesis and lysates were collected at the indicated time points. BMPR2 protein levels were quantified by Western blotting. Protein degradation rates were analyzed by nonlinear regression to determine half‐life values.

### Cell scratch test

Cells were grown to confluence and then incubated in serum‐free culture medium. They were treated with mitomycin (MCE) for 2 h before a single scratch was made across the monolayer using a sterile 10 μL pipette tip. One day later, the cells were fixed with 10% paraformaldehyde and stained with crystal violet for 15 min. Images of the scratches were captured with an Olympus IX71 inverted microscope (Olympus, Tokyo, Japan) and analyzed utilizing the Olympus CellSens Dimension software.

### Statistical analysis

All data are expressed as mean ± SEM from three or more independent experiments and analyzed with PASW Statistic 27 (SPSS Inc., Chicago, IL, USA). Data between two groups were compared using the Mann–Whitney U test. For more than two groups, the Kruskal–Wallis test was performed followed by Dunn's *post hoc* test. *P* < 0.05 was considered a significant difference.

## Results

### Increased DDX3X expression in PH endothelium

**Fig. 1:**
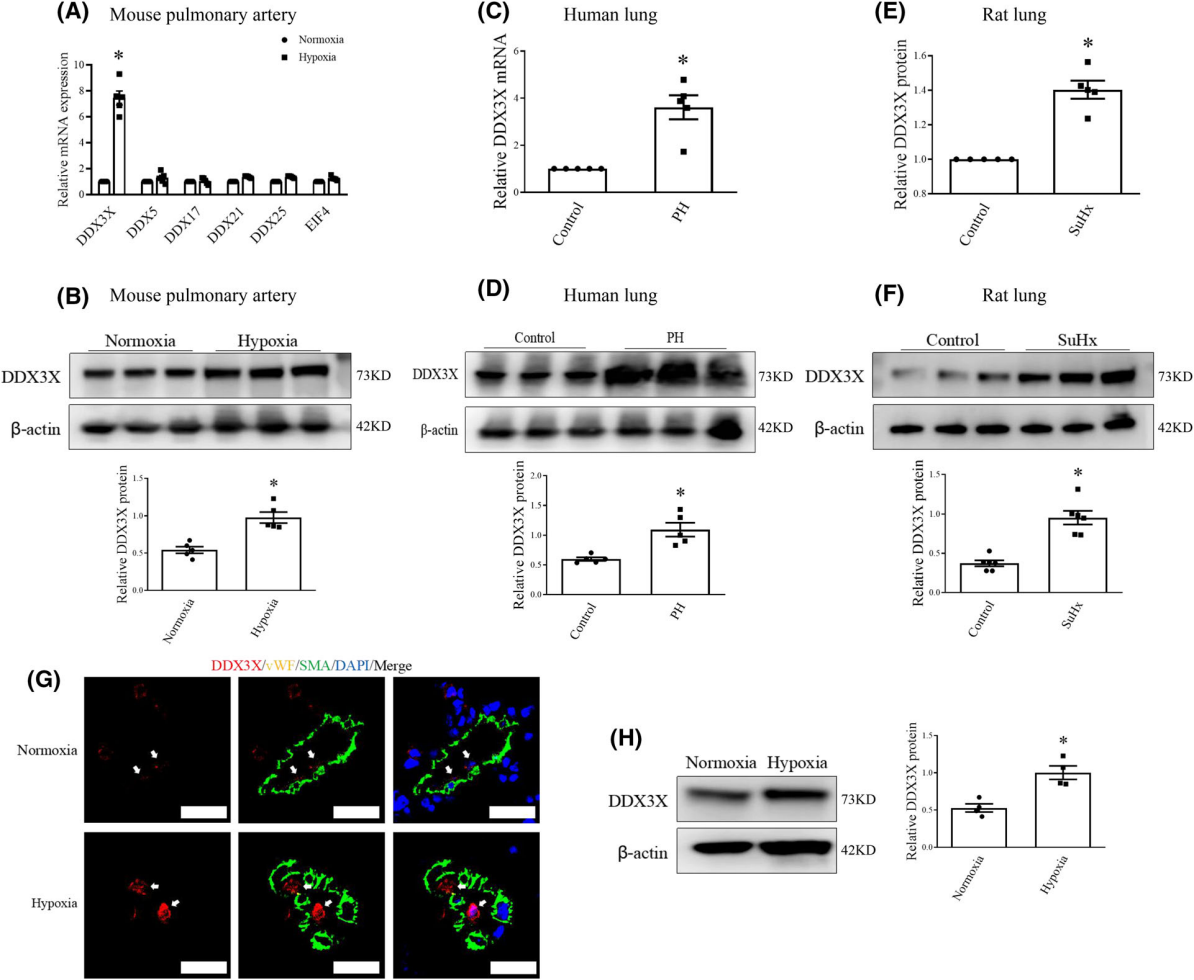
DDX3X Expression is increased in PH endothelium. (A) Expression pattern of DEAD‐box protein family in mouse pulmonary arteries was determined by qRT‐PCR. *n* = 5. (B) DDX3X protein expression in pulmonary arteries of hypoxia‐exposed mice and controls were evaluated by western blot analysis. *n* = 5. (C–F) DDX3X mRNA and protein levels in lung tissues of PH patients (C and D) and rats treated with sugen/hypoxia (SuHx; E and F) were evaluated by qRT‐PCR and western blot analysis, respectively. *n* = 5–6. (G) Representative immunofluorescent staining of DDX3X in pulmonary arteries of hypoxic PH mice and control (DDX3X, red fluorescence; vWF, yellow; SMA, green; 4′,6‐diamidino‐2‐phenylindole [DAPI], nuclei stain, blue; the white arrow indicated the location of DDX3X). Scale bars = 20 μm. (H) DDX3X protein levels in lung ECs from hypoxic PH mice and control were evaluated by western blot analysis. *n* = 4. Data are mean ± SEM; Data were analyzed using Kruskal‐Wallis test or Mann–Whitney *U*‐test; *P < 0.05. DDX3X, DEAD‐box protein 3 X‐linked; PH, pulmonary hypertension; qRT‐PCR, quantitative reverse transcription polymerase chain reaction; vWF, von Willebrand Factor; SMA, smooth muscle Actin; ECs, endothelial cells.

PH is characterized by EndoMT, which contributes to vascular remodeling by altering EC phenotype [4, 5]. Therefore, we first examined the changes in the expression profiles of DEAD‐box proteins in pulmonary arteries of experimental PH mice (Fig. 1A). Quantitative real‐time polymerase chain reaction (qRT‐PCR) analysis unveiled that among the DEAD‐box family members, DDX3X levels showed the most significant alteration in pulmonary arteries of PH mice compared to control vessels. This finding was confirmed by qRT‐PCR and western blot analyses on lung tissues from patients with PH and rodents exposed to chronic hypoxia, indicating a marked increase in DDX3X mRNA and protein levels, as compared with their corresponding controls (Fig. 1B–F).

Next, immunofluorescence staining was conducted to further assess the pattern of DDX3X expression in PH vessels (Fig. 1G). Of note, elevated DDX3X expression was primarily observed in the lung vascular intima of hypoxia‐challenged mice. Moreover, ECs isolated from hypoxic mice showed elevated DDX3X protein expression compared to those from normoxic mice (Fig. 1H). Thus, our results suggest that DDX3X expression is significantly upregulated in PH endothelium.

### DDX3X is positively correlated with EndoMT

**Fig. 2:**
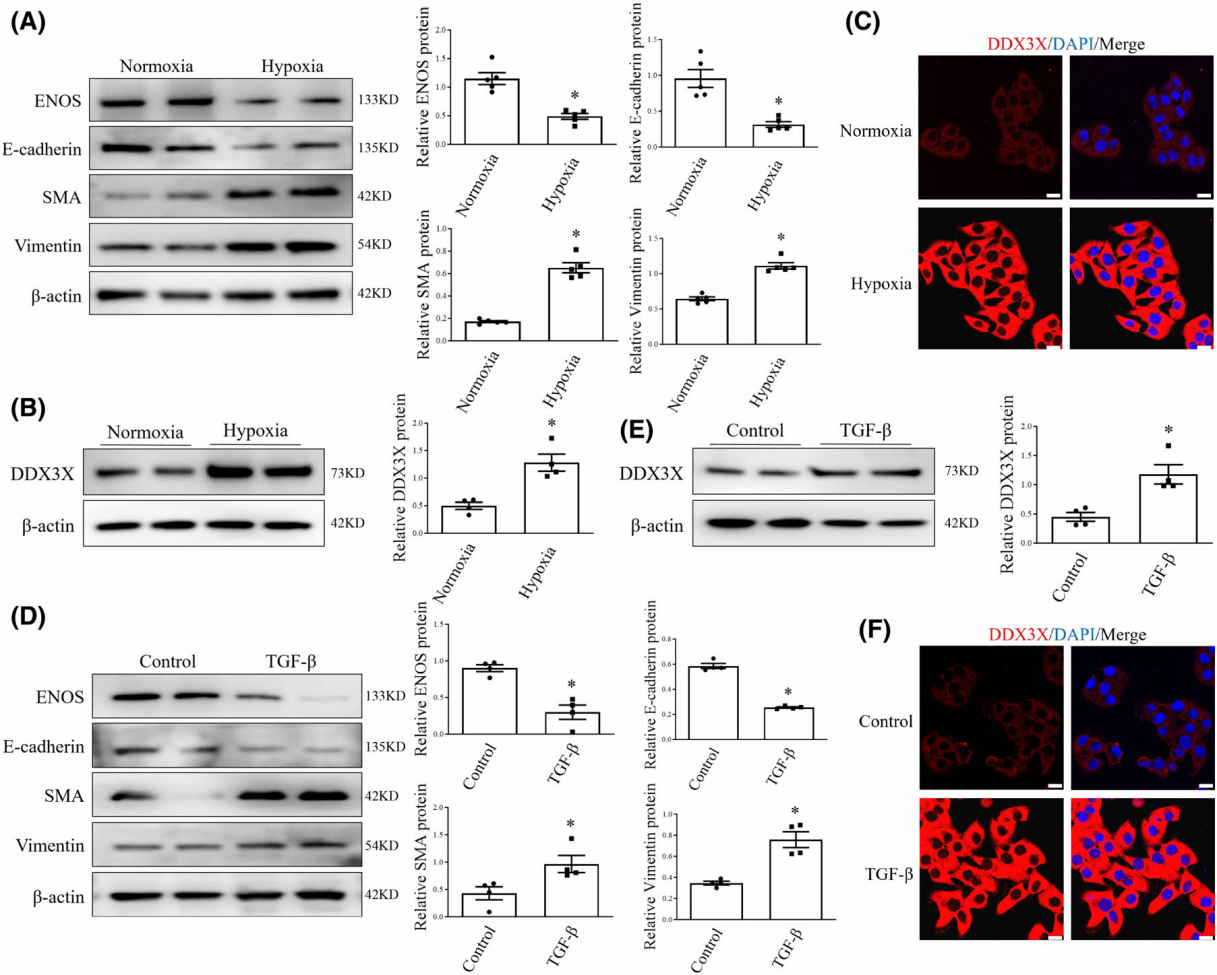
DDX3X level is elevated during EndoMT. (A) Protein levels of ENOS, E‐cadherin, SMA, and Vimentin in human lung ECs exposed to normoxia or hypoxia. *n* = 5. (B) ECs were exposed to normoxia or hypoxia. Cell lysates were obtained and probed with antibodies against DDX3X. *n* = 4. (C) Immunofluorescence confocal images revealed the effects of hypoxia on DDX3X (red) expression in ECs. Cell nuclei were stained with DAPI (blue). Scale bar = 20 μm. (D) ECs were treated with either vehicle or TGF‐β for 24 h. Expression of ENOS, E‐cadherin, SMA, and Vimentin protein was measured by western blot. *n* = 4. (E) DDX3X protein levels in ECs treated with either vehicle or TGF‐β. *n* = 4. (F) Representative immunofluorescent staining of DDX3X in ECs treated with either vehicle or TGF‐β (DDX3X, red fluorescence; nuclei stain, blue). Scale bars = 20 μm. Data are mean ± SEM; Data were analyzed using Mann–Whitney *U*‐test; *P < 0.05. EndoMT, endothelial‐to‐mesenchymal transition; ENOS, endothelial nitric oxide synthase; DAPI, 4′,6‐diamidino‐2‐phenylindole; TGF‐β, transforming growth factor‐beta.

To verify the *in vivo* findings, human pulmonary artery ECs were exposed to 1% oxygen for 24 h. Western blot analysis demonstrated that hypoxia triggered notable EndoMT, as evidenced by a loss of endothelial markers and an increase in mesenchymal markers, along with endothelial dysfunction (Fig. 2A). Notably, hypoxia stimulation simultaneously increased DDX3X protein levels in human lung ECs (Fig. 2B). Accordingly, the immunofluorescence study revealed intensified DDX3X signaling in ECs exposed to hypoxia (Fig. 2C).

Next, we investigated the expression pattern of DDX3X in an alternative model of EndoMT induced by transforming growth factor beta (TGF‐β) (Fig. 2D). Similarly, incubation with TGF‐β induced pronounced EndoMT in ECs and significantly increased DDX3X protein expression (Fig. 2E,F). Collectively, these data indicate a positive correlation between DDX3X and EndoMT.

### DDX3X promotes EndoMT

**Fig. 3:**
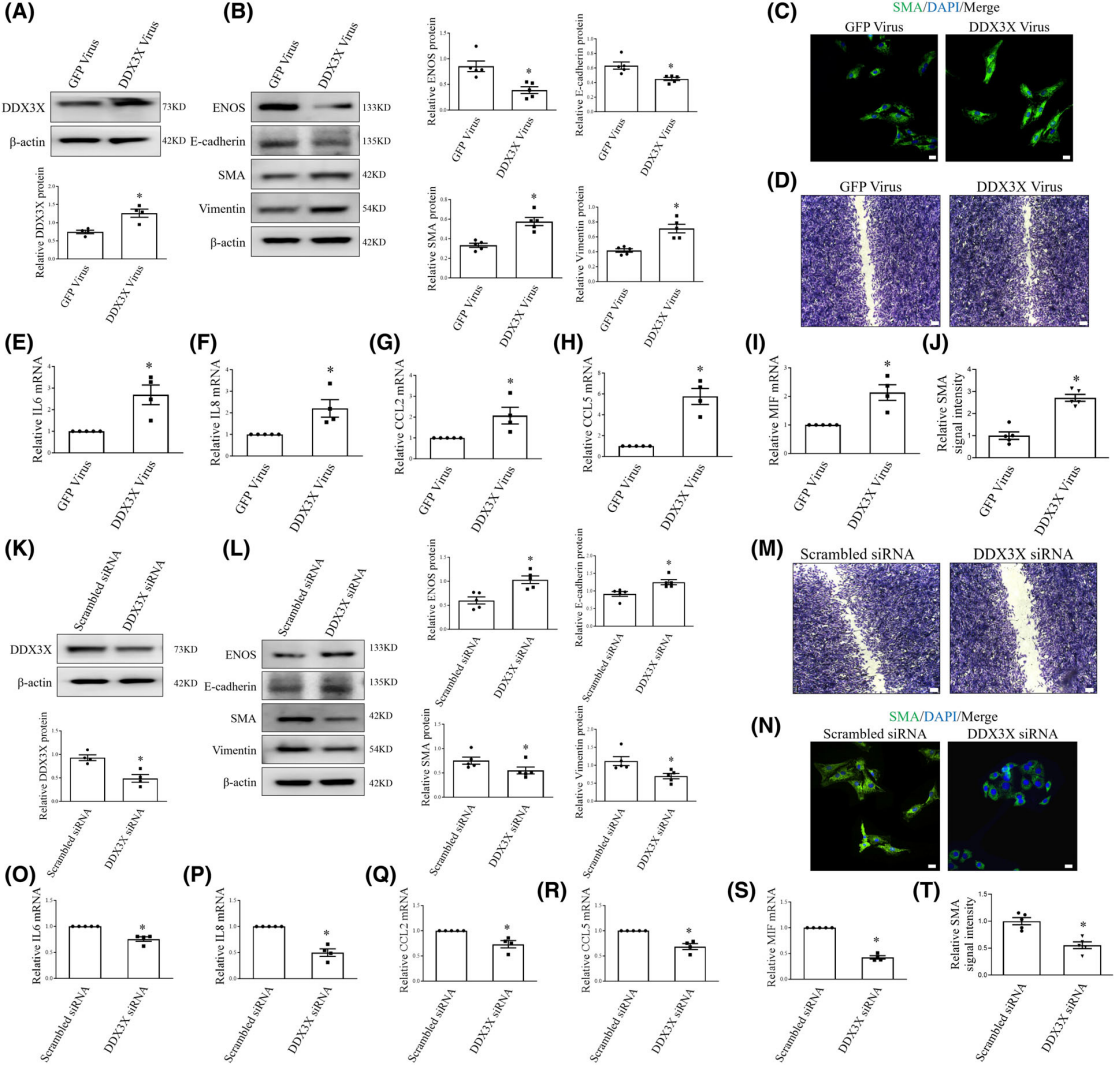
DDX3X induces mesenchymal transition of ECs. (A) DDX3X expression in ECs infected with GFP or DDX3X virus was assessed by western Blot analysis. *n* = 4. (B) Representative immunoblots of ENOS, E‐cadherin, SMA, and Vimentin protein expression in control or DDX3X overexpressing ECs. *n* = 5. (C) Confocal immunofluorescence imaging of SMA (green pseudocolor) in control and DDX3X‐overexpressing ECs. Cell nuclei were stained with DAPI (blue). Scale bar = 20 μm. (D) Cell migration in ECs infected with control or DDX3X virus. (E–I) IL‐6 (E), IL‐8 (F), CCL2 (G), CCL5 (H), and MIF (I) mRNA levels in control or DDX3X overexpressing ECs. *n* = 5. (J) Relative SMA signal intensity in control and DDX3X‐overexpressing ECs. (K) Protein levels of DDX3X in ECs infected with scrambled or DDX3X siRNA were assessed by western Blot analysis. *n* = 4. (L) Protein levels of ENOS, E‐cadherin, SMA, and Vimentin in human ECs infected with scrambled or DDX3X siRNA. *n* = 5. (M) The effects of DDX3X siRNA on EC migration were evaluated by scratch test. *n* = 3. (N) Immunofluorescence confocal images revealed SMA (green pseudocolor) in human ECs infected with scrambled or DDX3X siRNA. Cell nuclei were stained with DAPI (blue). Scale bar = 20 μm. (O–S) IL‐6 (O), IL‐8 (P), CCL2 (Q), CCL5 (R), and MIF (S) mRNA levels in ECs infected with scrambled or DDX3X siRNA. *n* = 5. (T), Relative SMA signal intensity in ECs infected with scrambled or DDX3X siRNA. Data are mean ± SEM; Data were analyzed using Mann–Whitney *U*‐test; *P < 0.05. GFP, green fluorescence protein.

To further clarify the contribution of DDX3X to endothelial homeostasis, lentiviral vectors carrying a DDX3X‐GFP construct allowing for co‐expression of DDX3X and a GFP reporter from independent promoters were applied to specifically upregulate DDX3X in HUVECs. Cells infected with a lentiviral vector containing a GFP construct served as a control. Overexpression of DDX3X was associated with elevated mesenchymal markers and decreased endothelial markers, as shown by western blot (Fig. 3A,B). Immunofluorescence staining revealed that ectopic DDX3X expression significantly increased SMA protein levels and induced a mesenchymal‐like morphological change in endothelial cells (Fig. 3C,J). Furthermore, DDX3X overexpression promotes EC migration and inflammation (Fig. 3D–I).

Next, we investigated the effects of DDX3X inhibition on EC phenotypic transition. In these experiments, human ECs were infected with either small interfering RNA (siRNA) targeting DDX3X or scrambled control. In accordance, silencing DDX3X inhibited EndoMT, endothelial migration, and inflammation (Fig. 3K–T). These findings underscore the critical role of DDX3X in regulating EC homeostasis.

### BMPR2 signaling in ECs is repressed by DDX3X

**Fig. 4:**
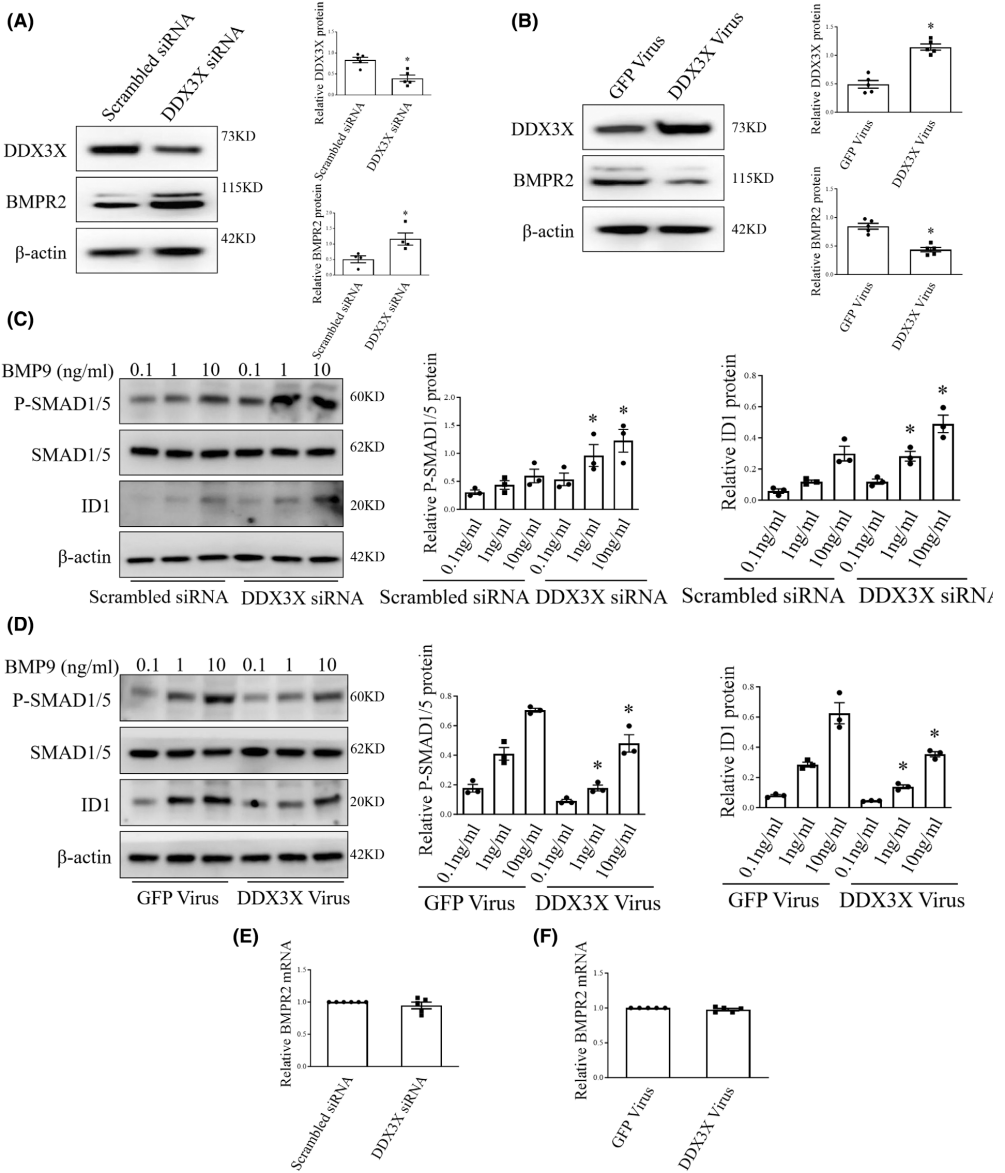
DDX3X decreases BMPR2 expression in EC. (A) DDX3X and BMPR2 protein levels in ECs infected with scrambled or DDX3X siRNA. *n* = 4. (B) Representative blots of DDX3X and BMPR2 protein in control or DDX3X overexpressing ECs. *n* = 5. (C, D) ECs were cultured with indicated concentrations of BMP9 (bone morphogenetic protein 9) for 1 h, and effects of DDX3X siRNA (C) DDX3X Virus (D) or appropriate controls on pSmad1/5 (phosphorylated Smads 1 and 5) over total Smad1/5 and Id1 (inhibitor of DNA binding 1) concentrations in ECs were determined by western blot. *n* = 3. (E) qRT‐PCR analysis of BMPR2 gene expression in human ECs infected with scrambled or DDX3X siRNA. *n* = 5. (F) BMPR2 mRNA level in ECs infected with control or DDX3X virus. *n* = 5. Data are mean ± SEM; Data were analyzed using Mann–Whitney *U*‐test or Kruskal‐Wallis test; *P < 0.05. BMPR2, bone morphogenetic protein receptor type 2; siRNA, small interfering RNA.

Based on the observed promotive effects of DDX3X on EndoMT, we proceeded to investigate the underlying mechanisms involved. BMPR2 represents the most common single culprit gene implicated in PH, and its dysfunction has recently been linked to EndoMT [24, 25]. Accordingly, we showed a reduction in BMPR2 protein levels in HUVECs exposed to hypoxia or TGF‐β (Fig. S1A,B), and inhibition of BMPR2 led to mesenchymal transition in these cells (Fig. S2A,B). Next, we examined whether the observed effects of DDX3X on ECs were associated with altered BMPR2 signaling (Fig. 4A,B). Notably, depleting DDX3X with siRNA increased BMPR2 levels in human ECs, while its overexpression suppressed BMPR2 protein expression. Consistently, BMPR2 signaling through Smad1/5 was reduced in ECs infected with DDX3X lentivirus, whereas DDX3X silencing increased phospho‐Smad1/5 and Id1 protein levels, as compared with their controls (Fig. 4C,D). Nevertheless, BMPR2 mRNA levels in ECs were unaffected by either DDX3X knockdown or overexpression, suggesting that DDX3X might directly modulate BMPR2 protein levels (Fig. 4E,F).

### DDX3X interacts with BMPR2

**Fig. 5:**
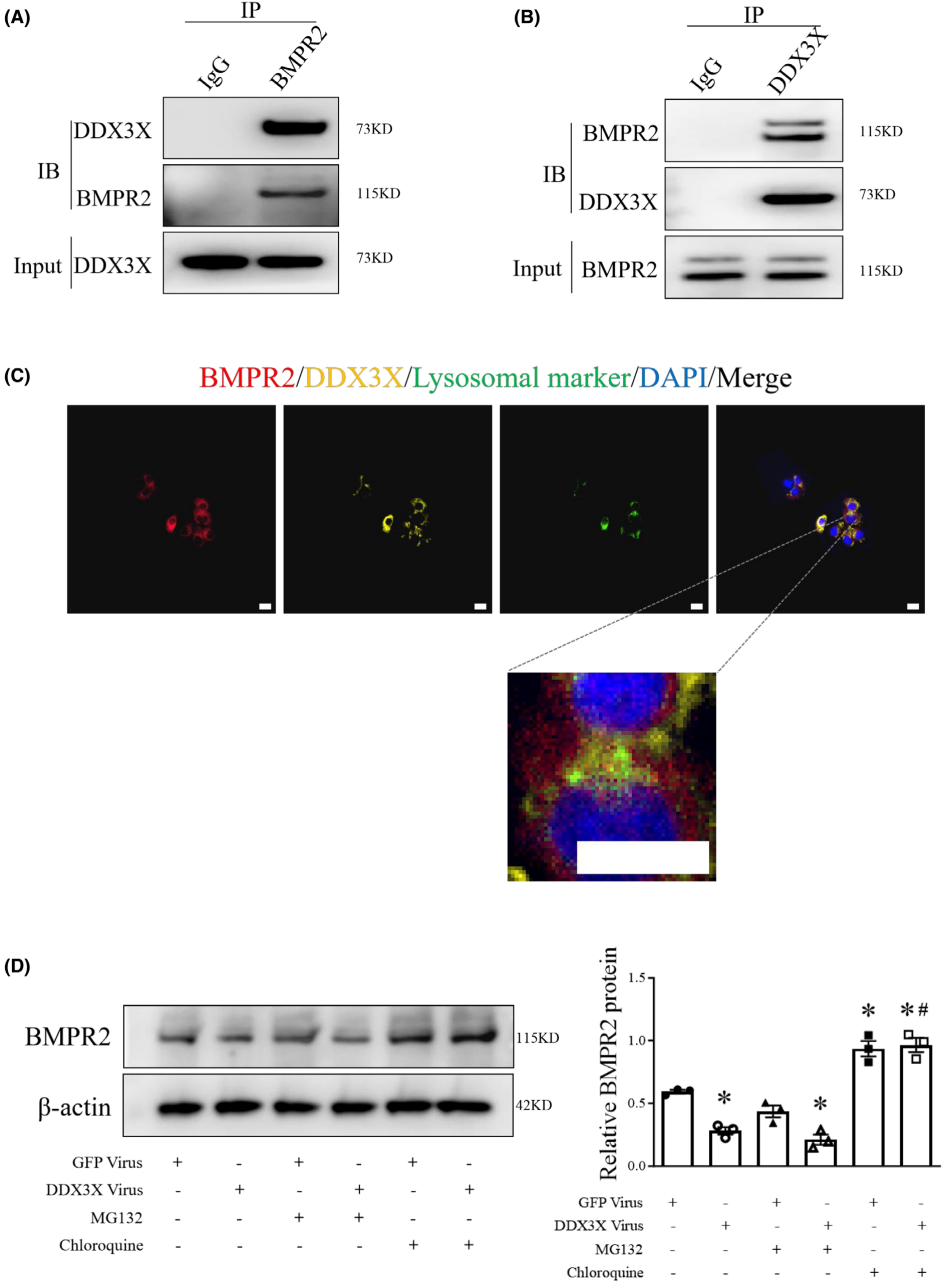
DDX3X associates with and promotes lysosomal degradation of BMPR2. (A, B) Coimmunoprecipitation assay of DDX3X and BMPR2 in ECs. (C) Immunofluorescence analysis reveals colocalization of DDX3X (yellow), BMPR2 (red), and lysosomal marker (green, Lyso‐Tracker Green) in human ECs. Scale bar = 20 μm. (D) BMPR2 protein expression in control or DDX3X overexpressing ECs in the presence or absence of either MG132 or Chloroquine was determined by western blot. *n* = 3. Data are mean ± SEM; Data were analyzed using the Kruskal–Wallis test; *P < 0.05 compared with the corresponding GFP controls; #*P* > 0.05 compared with GFP virus plus chloroquine group.

To further validate the association between DDX3X and BMPR2, a co‐immunoprecipitation assay was performed using detergent extracts from HUVECs. As shown in Fig. 5A, western blot analysis of the immunoprecipitates revealed a specific DDX3X band that was captured by the BMPR2 antibody, but not by control IgG. Similarly, co‐immunoprecipitation with an anti‐DDX3X antibody confirmed the presence of BMPR2 in the DDX3X complex, indicating a potential molecular association between DDX3X and BMPR2 (Fig. 5B). Supporting this, immunofluorescence analysis showed the colocalization of DDX3X and BMPR2 protein (Fig. 5C). To assess the effect of DDX3X on BMPR2 protein stability, we conducted cycloheximide chase assays in control and DDX3X‐overexpressing ECs. Consistent with the impact of hypoxia, DDX3X overexpression led to a decrease in BMPR2 half‐life in ECs, as shown by western blot analysis (Figs S3 and S4). These data demonstrate that DDX3X overexpression accelerates BMPR2 degradation.

Prior researches have suggested that BMPR2 is degraded via the lysosomal pathway [26, 27]. Chloroquine, a known lysosome inhibitor, was utilized to assess whether the suppression of BMPR2 by DDX3X is dependent on the lysosomal system. Our data demonstrated that DDX3X‐mediated downregulation of BMPR2 protein was partially rescued by chloroquine treatment, but not the proteasome inhibitor—MG132, indicating that DDX3X likely facilitates the lysosomal degradation of BMPR2 (Fig. 5D). In addition, this lysosome‐dependent degradation mechanism was further validated using Bafilomycin A1, another lysosomal inhibitor, which similarly attenuated the DDX3X‐mediated reduction in BMPR2 (Fig. S5). Immunofluorescence costaining of DDX3X, BMPR2, and lysosomal marker in ECs revealed partial colocalization among DDX3X, BMPR2, and lysosomal marker. Additionally, colocalization of DDX3X and BMPR2 was observed outside of lysosomes, suggesting that DDX3X might facilitate the transport of BMPR2 to lysosomes (Fig. 5C).

DEAD‐box proteins are well known for their roles in modulating RNA‐related biological processes. To investigate whether the ATPase and helicase activities of DDX3X are essential for its regulation of BMPR2 degradation, we generated ATPase‐ and helicase‐deficient DDX3X mutants based on conserved motifs in the DEAD‐box family, as previously reported [28]. Interestingly, similar to wild‐type DDX3X, DDX3X mutants reduced BMPR2 levels and promoted the loss of endothelial markers along with the acquisition of mesenchymal traits, suggesting that the ATPase and helicase activities of DDX3X are dispensable for its regulation of BMPR2 expression and EC transition (Fig. S6A,B). Taken together, these findings implicate that BMPR2 signaling might act as a downstream mediator in DDX3X‐induced EndoMT.

### DDX3X facilitates EndoMT via a BMPR2‐dependent pathway

**Fig. 6:**
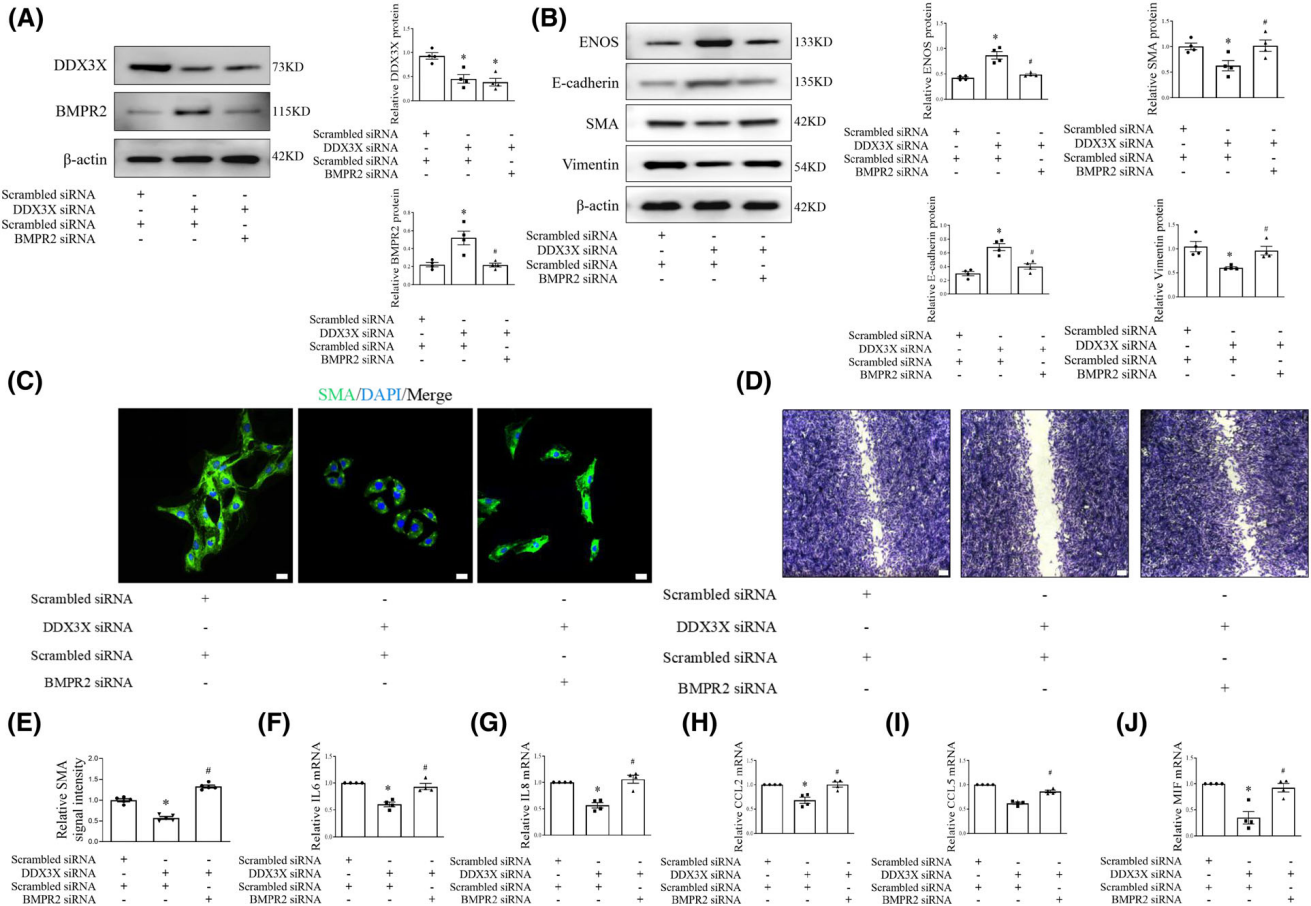
DDX3X triggers EndoMT by disrupting BMPR2 signaling. (A) Representative immunoblots of DDX3X and BMPR2 in ECs infected with DDX3X siRNA receiving either scrambled or BMPR2 siRNA. *n* = 3. (B) Representative immunoblots of ENOS, E‐cadherin, SMA, and Vimentin protein in ECs infected with DDX3X siRNA receiving either scrambled or BMPR2 siRNA. (C) SMA (green pseudocolor) immunofluorescence in human ECs treated with DDX3X siRNA plus scrambled or BMPR2 siRNA. Cell nuclei were stained with DAPI (blue). Scale bar = 20 μm. (D) Cell migration in ECs infected with DDX3X siRNA receiving either scrambled or BMPR2 siRNA. (E) Relative SMA signal intensity in human ECs treated with DDX3X siRNA plus scrambled or BMPR2 siRNA. (F–J), IL‐6 (F), IL‐8 (G), CCL2 (H), CCL5 (I), and MIF (J) mRNA levels in ECs infected with DDX3X siRNA receiving either scrambled or BMPR2 siRNA. *n* = 4. Data are mean ± SEM; Data were analyzed using the Kruskal–Wallis test; *P < 0.05 vs scrambled siRNA, ^#^ *P* < 0.05 vs BMPR2 siRNA.

To better understand how BMPR2 influences the effects of DDX3X on EndoMT, HUVECs were infected with DDX3X siRNA alongside either scrambled or BMPR2 siRNA. Notably, we found that BMPR2 siRNA blocked the protective effects of DDX3X silencing on EC homeostasis, as demonstrated by western blot and immunofluorescence analyses (Fig. 6A,B). In addition, DDX3X siRNA transfection‐mediated repression of EC migration, dysfunction, and inflammation was partially reversed by BMPR2 knockdown, suggesting that DDX3X‐induced regulation of ECs might be mediated through BMPR2 suppression (Fig. 6C–J). In summary, our data indicate that DDX3X promotes EndoMT by interacting with and enhancing the lysosomal degradation of BMPR2.

## Discussion

Although prior studies have suggested an essential role of RNA helicases in vascular abnormalities, their effects on endothelial homeostasis remain elusive [18, 22, 29]. This study might be the first to show that DDX3X, a key member of the DEAD‐box RNA helicase family, negatively impacts human endothelium by promoting aberrant mesenchymal transition, which is fundamental to various vascular disorders and closely linked to EC function [22, 30]. Mechanistically, we identified a novel pathway that goes beyond the primary RNA unwinding capability of DDX3X, wherein it promotes endothelial transition by binding to BMPR2 protein and accelerating its lysosomal degradation, consequently interrupting the downstream signaling of BMPR2 [26, 27]. Therefore, targeting DDX3X in ECs might represent a viable strategy for maintaining endothelial homeostasis.

The DEAD‐box protein family, the largest group of RNA helicases, is pivotal for regulating multiple biological processes involving vascular cells [18, 31]. Our prior study demonstrates that DDX5 attenuates macrophage migration inhibitory factor‐elicited smooth muscle dedifferentiation [32]. Moreover, DDX3X has been reported to promote the development of aneurysms by inducing vascular smooth muscle cell transformation [28]. Nevertheless, despite the regulatory role of DEAD‐box proteins in vascular cell behavior, their involvement in the regulation of endothelial cell homeostasis remains poorly understood.

In this study, DDX3X is identified as the DEAD‐box family member displaying the most pronounced elevation in lung vessels of rodent PH models, primarily localized to the intima. In PH, ECs undergo mesenchymal transition characterized by loss of endothelial markers and acquisition of mesenchymal traits, facilitating vascular remodeling by exacerbating endothelial dysfunction and inflammation [4, 9]. Therefore, we first evaluated the changes in DDX3X levels during EC phenotypic transition, revealing its upregulation in both hypoxia and TGF‐β‐triggered EndoMT processes. Of note, targeted repression of DDX3X using specific siRNA impedes the expression of EndoMT‐associated genes in ECs, whereas overexpression of DDX3X promotes their transformation into a mesenchymal state. Supporting this, DDX3X induces endothelial inflammation and dysfunction that are commonly associated with cell transition, thereby reinforcing the role of DDX3X in EC phenotypic switching and prompting us to further explore the underlying mechanisms [5, 7].

Heterozygous loss‐of‐function germline mutations in the BMPR2 gene are the most common genetic cause of heritable pulmonary arterial hypertension, accounting for 75% of pulmonary arterial hypertension cases in families [24, 25, 33]. Importantly, BMPR2 plays a crucial role in maintaining EC function and inhibiting the EndoMT process, as its dysfunction or mutation can transition endothelium to mesenchymal‐like cells [4, 34, 35]. Notably, we demonstrated that DDX3X decreased BMPR2 protein expression, and its silencing further elevated BMPR2 levels. Consistently, BMPR2 signaling via Smad1/5 is inhibited in ECs with DDX3X overexpression, while Smad1/5 phosphorylation and Id1 expression are elevated following DDX3X depletion, indicating that DDX3X might negatively influence BMPR2 signaling [36, 37, 38].

Intriguingly, silencing or overexpressing DDX3X has no effect on BMPR2 mRNA levels, despite its established role as an mRNA modulator [22, 39]. Through co‐immunoprecipitation, we identify a novel interplay between DDX3X and BMPR2 protein, which is further validated by immunofluorescence assays showing colocalization of these two molecules. In addition, our data indicate that DDX3X is involved in enhancing lysosomal degradation of BMPR2 protein. Notably, transfection with BMPR2 siRNA counteracted the protective effects of DDX3X depletion on endothelial homeostasis, dysfunction, and inflammation, indicating that DDX3X‐mediated modulation of endothelial cell phenotype is, at least in part, achieved through disruption of BMPR2 signaling.

## Limitation

This study has several limitations. First, currently, there are no specific antagonists targeting DDX3X, which hinders our ability to validate the translational value of DDX3X inhibition in EndoMT‐related vascular diseases. Second, there were no relevant animal studies that examine the direct influence of DDX3X on EC transition. Future studies are needed to explore the *in vivo* regulatory role of DDX3X in EC homeostasis.

## Conclusions

In conclusion, our study points to a novel role for DDX3X in controlling EC mesenchymal transition through its interaction with and degradation of BMPR2. DDX3X might serve as a potential therapeutic target in vascular abnormalities related to EndoMT.

## Conflicts of interest

The authors declare no conflict of interest.

## Author contributions

YZ, JW, DHQ, and YFL contributed equally to this study. DPW, JZ, and YF designed the experiments. YZ, JW, DHQ, YFL, TLC, and JZ performed the experiments. YZ and DHQ analyzed data. JZ and YF wrote the manuscript and made manuscript revisions. DPW supervised the study. All authors read and approved the final version of this manuscript.

## Supporting information


**Fig. S1.** Protein levels of BMPR2 in ECs exposed to hypoxia or TGF‐β.
**Fig. S2.** Protein levels of BMPR2, ENOS, E‐cadherin, SMA, and Vimentin in human ECs transfected with scrambled or BMPR2 siRNA.
**Fig. S3.** Half‐life of BMPR2 in ECs under normoxia and hypoxia.
**Fig. S4.** Half‐life of BMPR2 in ECs transfected with GFP or DDX3X virus.
**Fig. S5.** Protein levels of BMPR2 in ECs transfected with GFP or DDX3X virus and treated with 10 nmoL·mL^−1^ of Bafilomycin A1.
**Fig. S6.** Protein levels of BMPR2, ENOS, E‐cadherin, SMA, and Vimentin in ECs transfected with control or DDX3X mutant plasmids.

**Table S1.** Specific sequences of the primers.
**Table S2.** Antibodies for immunoblot analyses.

## Data Availability

The data are available from the corresponding author upon reasonable request.
