# Supplementary material for: DDX3X induces mesenchymal transition of endothelial cells by disrupting BMPR2 signaling
Source: FEBS Open Bio. 2025 Nov 4;16(4):737–50. doi: 10.1002/2211-5463.70155 (PMC13042637; doi:10.1002/2211-5463.70155)
Supplement: Supplementary file 1 — Fig. S1. Protein levels of BMPR2 in ECs exposed to hypoxia or TGF‐β. Fig. S2. Protein levels of BMPR2, ENOS, E‐cadherin, SMA, and Vimentin in human ECs transfected with scrambled or BMPR2 siRNA. Fig. S3. Half‐life of BMPR2 in ECs under normoxia and hypoxia. Fig. S4. Half‐life of BMPR2 in ECs transfected with GFP or DDX3X virus. Fig. S5. Protein levels of BMPR2 in ECs transfected with GFP or DDX3X virus and treated with 10 nmoL·mL−1 of Bafilomycin A1. Fig. S6. Protein levels of BMPR2, ENOS, E‐cadherin, SMA, and Vimentin in ECs transfected with control or DDX3X mutant plasmids. [file FEB4-16-737-s001.pdf]

Supplemental Figure 1

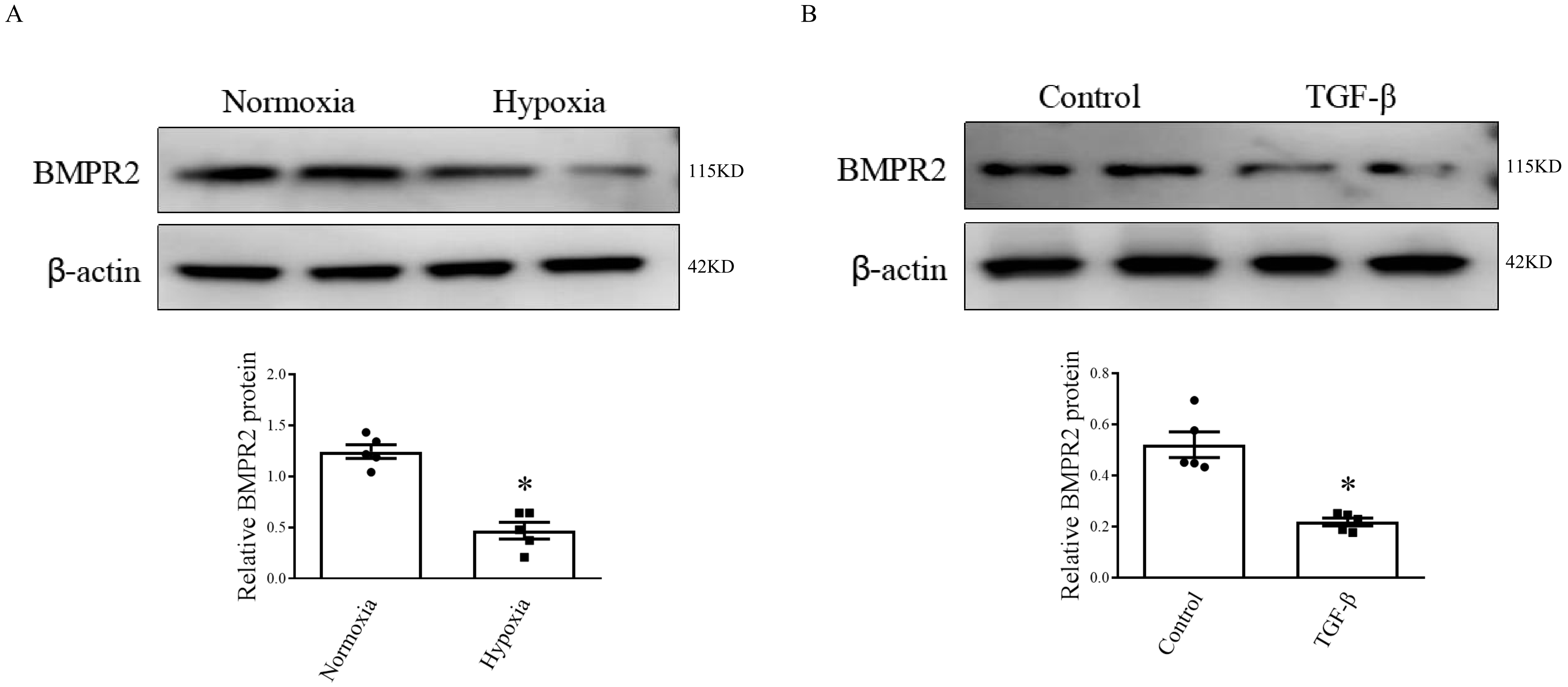

Supplemental figure 1. **A** and **B**, Protein levels of BMPR2 in ECs exposed to hypoxia or TGF-β were assessed by Western Blot analysis. n=5. Data are mean ± SEM; Data were analysed using Mann-Whitney U-test; *P<0.05.

Supplemental Figure 2

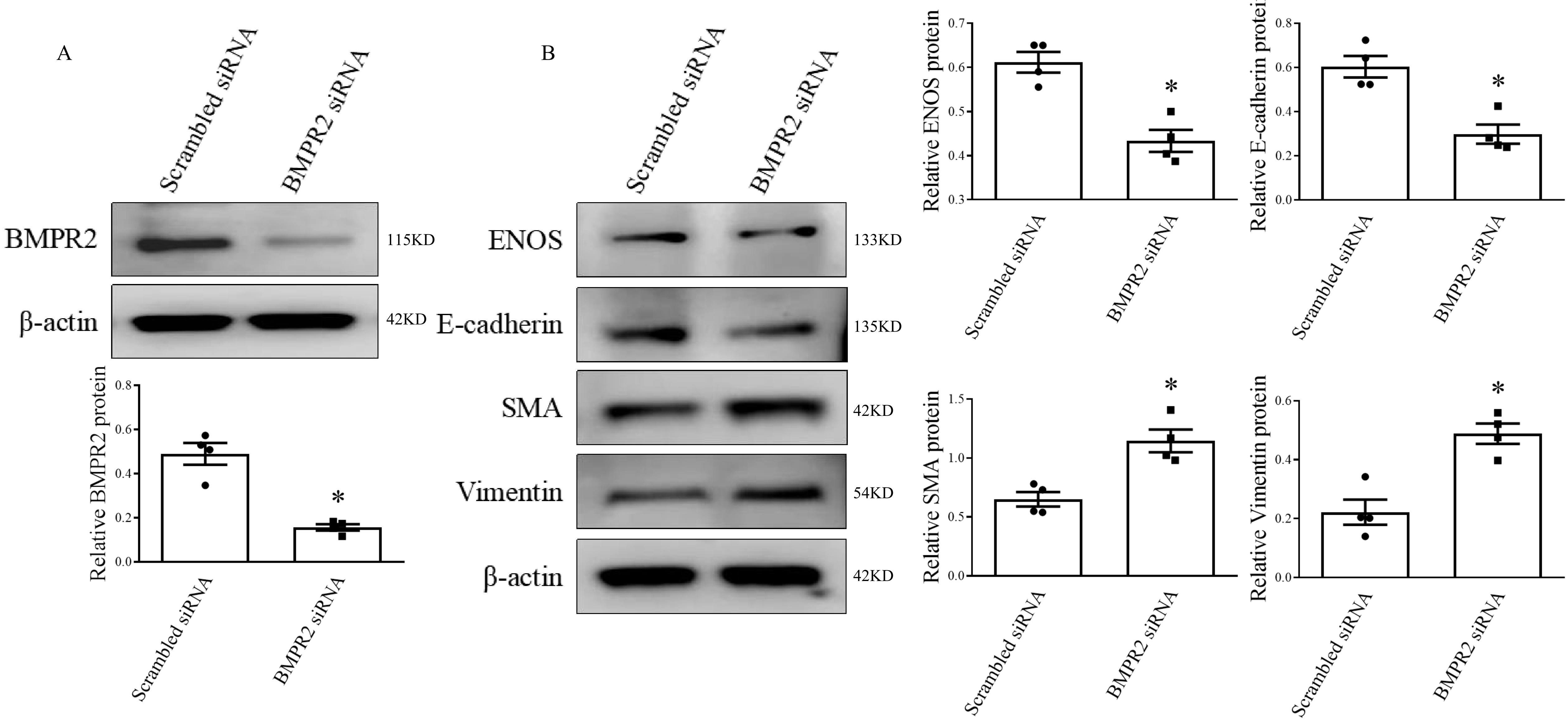

Supplemental figure 2. **A**, BMPR2 expression in ECs transfected with scrambled or BMPR2 siRNA were assessed by Western Blot analysis. n=4. **B**, Protein levels of ENOS, E-cadherin, SMA, and Vimentin in human ECs transfected with scrambled or BMPR2 siRNA. n=4. Data are mean ± SEM; Data were analysed using Mann-Whitney U-test; *P<0.05.

Supplemental Figure 3

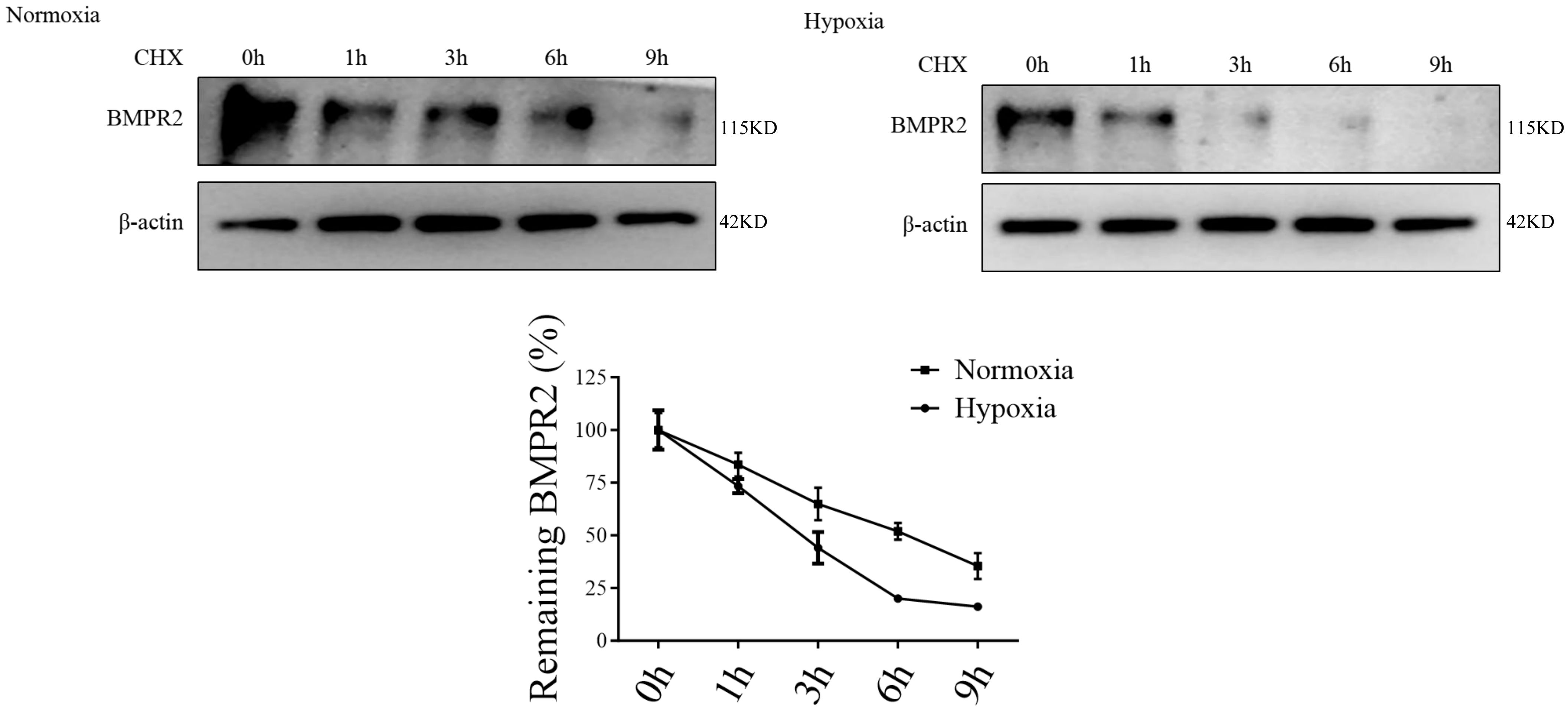

Supplemental figure 3. Half-life of BMPR2 in ECs under normoxia and hypoxia. Cells were treated with cycloheximide (10 mg/ml) for the indicated times, and BMPR2 abundance was determined by Western blot. n = 3.

Supplemental Figure 4

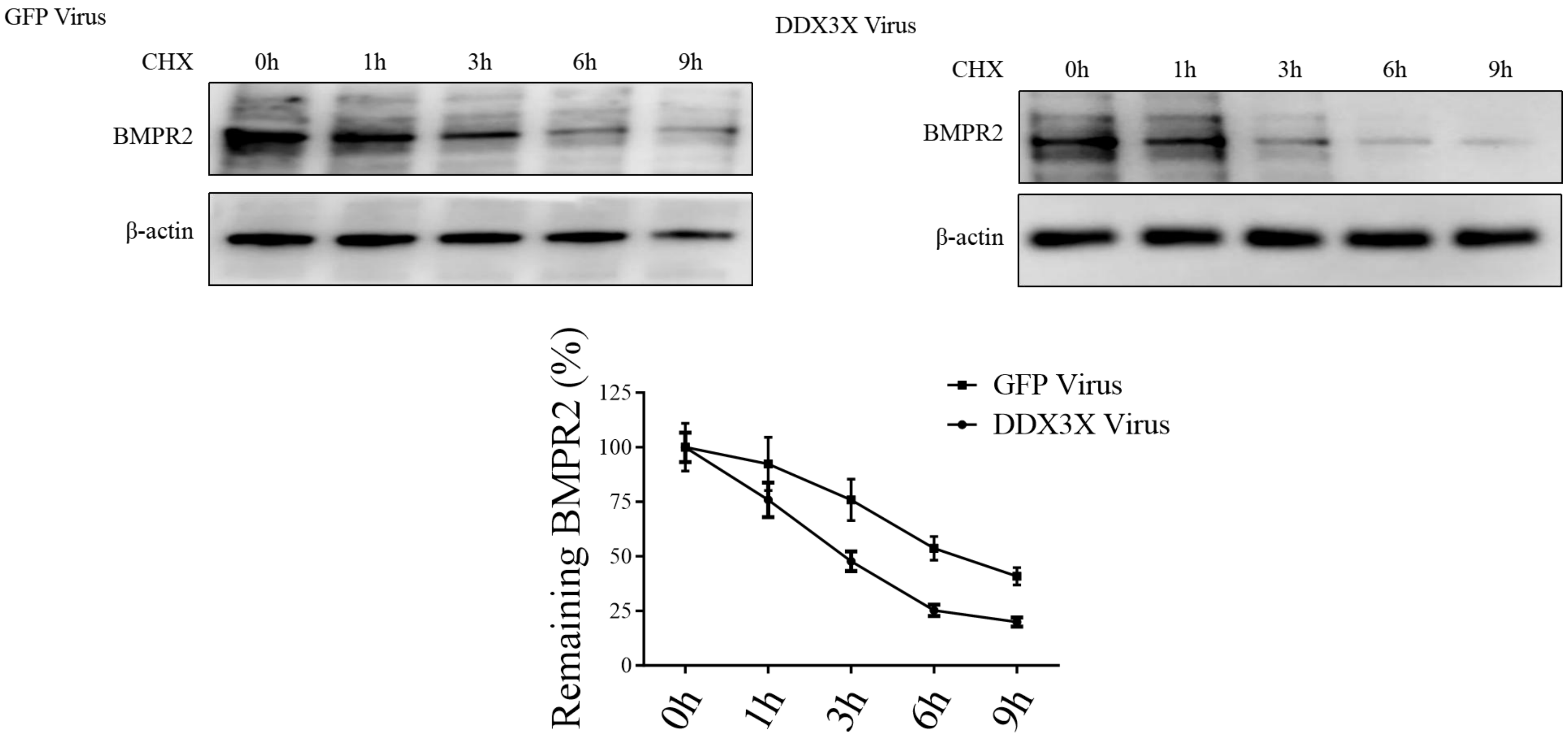

Supplemental figure 4. Half-life of BMPR2 in ECs transfected with GFP or DDX3X virus. Cells were treated with cycloheximide (10 mg/ml) for the indicated times, and BMPR2 abundance was determined by Western blot. n = 3.

Supplemental Figure 5

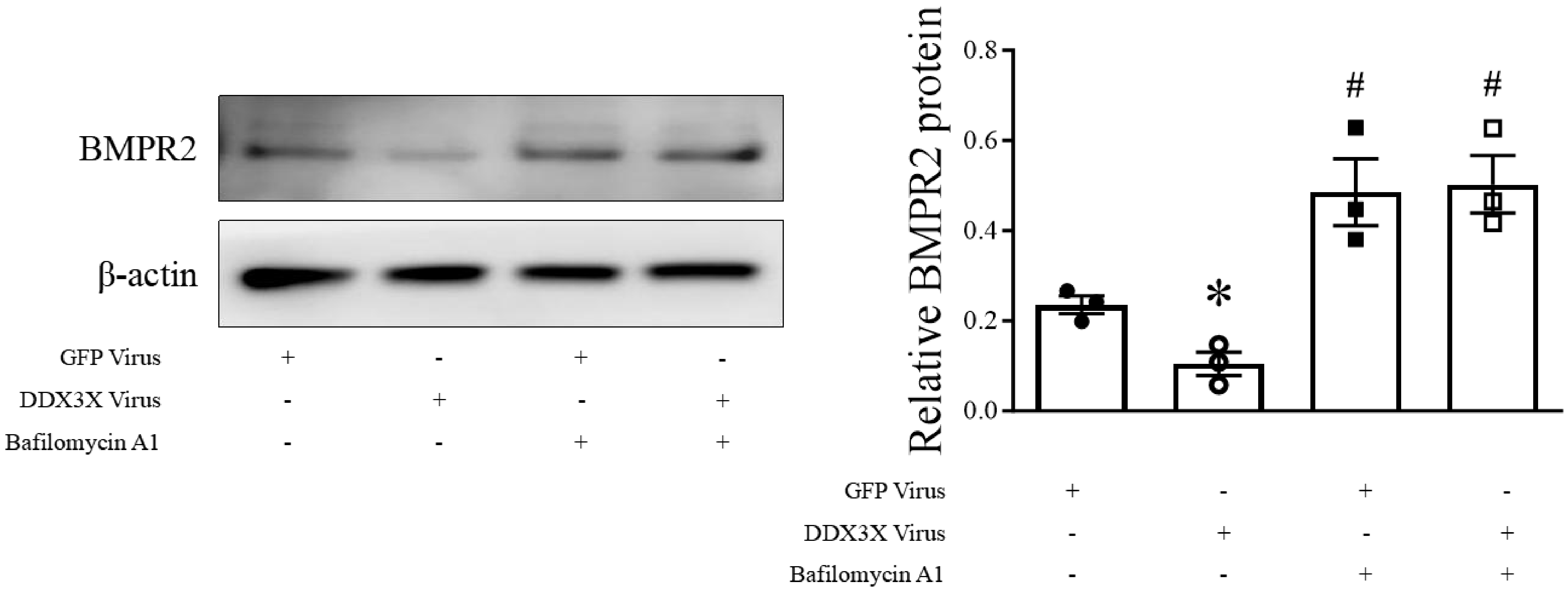

Supplemental figure 5. ECs transfected with GFP or DDX3X virus were treated with 10 nmol/ml of Bafilomycin A1, and protein concentrations of BMPR2 were determined. n=3. Data are mean ± SEM; Data were analysed using Mann-Whitney U-test; *P<0.05.

Supplemental Figure 6

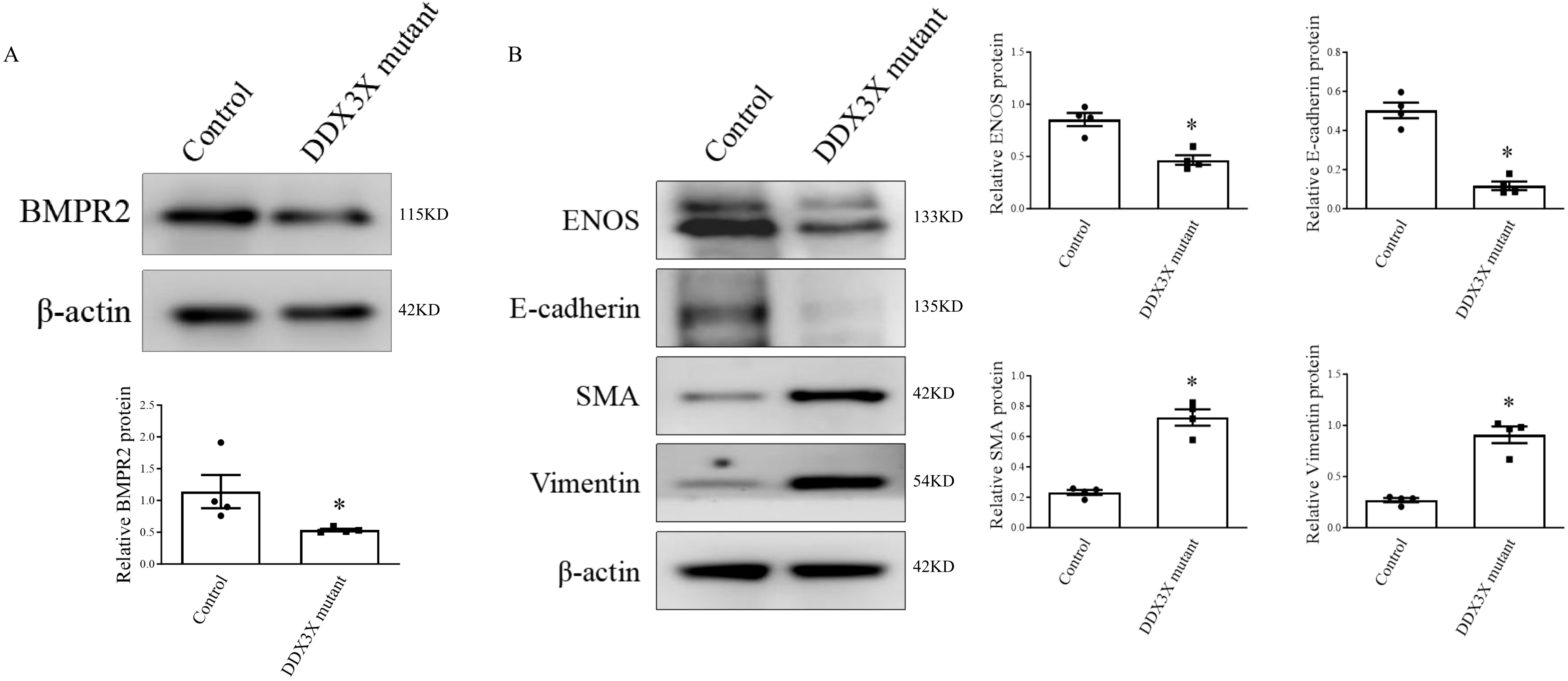

Supplemental figure 6. **A**, Protein levels of BMPR2 in ECs transfected with control or DDX3X mutant plasmids. n=3. **B**, Protein levels of ENOS, E-cadherin, SMA, and Vimentin in ECs transfected with control or DDX3X mutant plasmids. n=4. Data are mean ± SEM; Data were analysed using Mann-Whitney U-test; *P<0.05.
