# Supplementary material for: DDX3X induces mesenchymal transition of endothelial cells by disrupting BMPR2 signaling
Source: FEBS Open Bio. 2025 Nov 4;16(4):737–50. doi: 10.1002/2211-5463.70155 (PMC13042637; doi:10.1002/2211-5463.70155)
Supplement: Supplementary file 2 — Table S1. Specific sequences of the primers. Table S2. Antibodies for immunoblot analyses. [file FEB4-16-737-s002.docx]

**Supplement**

**Supplemental Table Ⅰ. Specific sequences of the primers**

| Primer name | Primer sequences |
| --- | --- |
| Human DDX3X | For: 5’ AGCAGTTTTGGATCTCGTAGTG 3’  Rev: 5’ ACTGTTTCCACCACGTTCAAAT 3’ |
| Human IL6 | For: 5’ TTAGTCCTTCCTACCCCAATTTCC 3’  Rev: 5’ TTGGTCCTTAGCCACTCCTTC 3’ |
| Human β-actin | For: 5’ GAGCTACGAGCTGCCTGACG 3’  Rev: 5’ GTAGTTTCGTGGATGCCACAG 3’ |
| Human IL8 | For: 5’ ACTGAGAGTGATTGAGAGTGGAC 3’  Rev: 5’ AACCCTCTGCACCCAGTTTTC 3’ |
| Human CCL2 | For: 5' CAGCCAGATGCAATCAATGCC 3'  Rev: 5' TGGAATCCTGAACCCACTTCT 3' |
| Human CCL5 | For: 5’ CCAGCAGTCGTCTTTGTCAC 3’  Rev: 5’ CTCTGGGTTGGCACACACTT 3’ |
| Human MIF | For: 5' GAGGGGTTTCTGTCGGAGC 3'  Rev: 5' GTTCGTGCCGCTAAAAGTCA 3' |

**Supplemental Table II. Antibodies for immunoblot analyses**

| Antibody | Manufacturer | Catalog | Source of species | MW (kDa) |
| --- | --- | --- | --- | --- |
| DDX3X | Proteintech | 11115-1-AP | rabbit | 73 |
| BMPR2 | Abcam | Ab130206 | mouse | 115 |
| eNOS | Proteintech | 27120-1-AP | rabbit | 133 |
| E-cadherin | CST | 14472 | mouse | 135 |
| SMA | Abcam | Ab7817 | mouse | 42 |
| Vimentin | Proteintech | 10366-1-AP | rabbit | 54 |
| β-actin | Proteintech | 66009-1-Ig | mouse | 42 |
| ID1 | Proteintech | 18475-1-AP | rabbit | 20 |
| Smad1/5 | Abcam | Ab300164 | rabbit | 62 |
| P-Smad1/5 | CST | 9516 | rabbit | 60 |
